# Leveling of arterial wall stiffness between aortic arch and left carotid artery due to aging is associated with reduced volume flow towards the brain: pulse wave velocity evaluation with high-field velocity-encoded MRI

**DOI:** 10.1186/1532-429X-15-S1-P248

**Published:** 2013-01-30

**Authors:** Eleanore Kroner, Hildo J Lamb, Pieter J van den Boogaard, Hans-Marc J Siebelink, Ernst E van der Wall, Albert de Roos, Jos J Westenberg

**Affiliations:** 1 LUMC, Den Haag, Netherlands; 2 Radiology, LUMC, Leiden, Netherlands

## Background

A discrete transition of wall stiffness at the interface of a compliant aorta and stiffer carotid arteries creates a reflection site for the aortic pulse wave and consequently limits excessive pulsatile energy transmission towards the brain. Arterial wall stiffness can be expressed by the pulse wave velocity (PWV) and dual-slice one-directional through-plane velocity-encoded (VE) MRI is well-validated for accurate PWV-assessment. The hypothesis is that with aging, leveling of PWV across the aortic arch and carotid artery will occur with negative effect on the flow towards the brain. The purpose of this study was to evaluate PWV and volume flow in aorta and carotid artery in younger and older volunteers using 3T VE MRI.

## Methods

**Figure 1:**
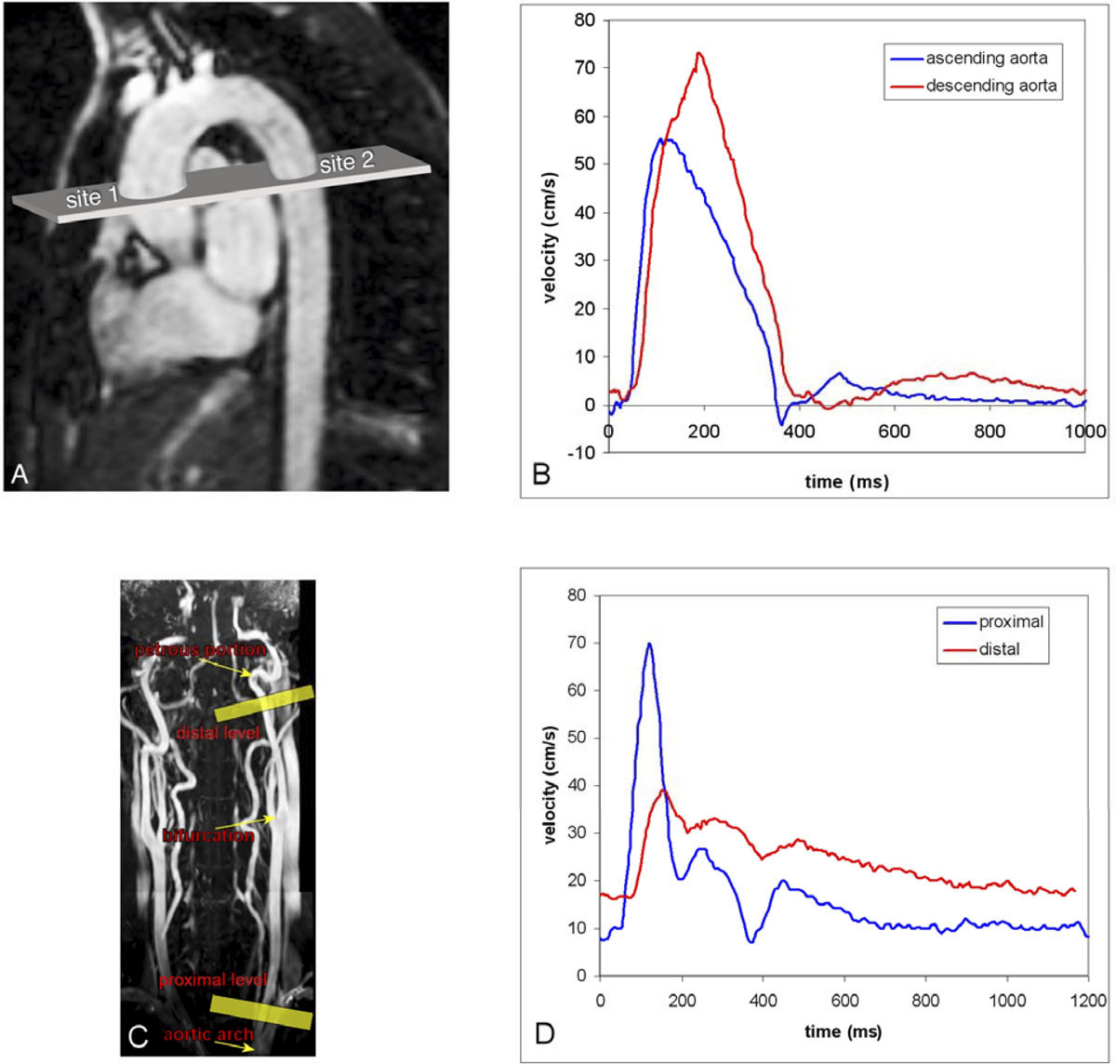
On a longitudional scout image of the aortic arch (1A), a single one-directional through-plane velocity-encoded MRI acquisition was planned perpendicular to the ascending and proximal descending aorta. Figure 1B represents the resulting flow velocity wave forms assessed at the two respective sites, illustrating the pulse wave propagation between sites. Figure 1C shows a coronal maximum intensity projection of 3D time-of-flight data of the carotid arteries, from the origin at the aortic arch to the circle of Willis. Two one-directional velocity-encoded MRI acquisitions were planned (yellow lines: one proximally at the left common carotid artery just above the aortic arch and one distally just below the petrous portion of the left internal carotid artery). Figure 1D represents the resulting velocity wave forms assessed at the two respective sites, illustrating the pulse wave propagation.

Sixteen younger volunteers with age<30 years (mean 25±3years) and sixteen older volunteers with age>45 years (mean 56±6years) underwent 3T MRI (Philips) to assess PWV in the aortic arch and the left carotid artery based on the transit-time method. PWV in the aortic arch was acquired from a single VE MRI acquisition (Figure 1A) and from wave propagation analysis on the velocity-time curves (Figure 1B). Path length (required for PWV calculation) was measured manually on a sagittal view of the aorta. Also cardiac output (stroke volume per heart beat) was determined from the flow curves. Carotid PWV was assessed by 2 VE MRI acquisitions, perpendicular to the left carotid artery (Figure 1C), one proximal at the origin of the common carotid artery and one distal in the internal carotid artery, just below the petrous portion. Path length (required for PWV calculation) was measured manually on maximal intensity projection a 3D time-of-flight MR angiogram. Stroke volume per heart beat was determined from the flow curves at both levels in the carotid artery.

## Results

All measurements are presented in Table 1. Older volunteers had significant longer aortic arch trajectory. PWV both in aortic arch as well as carotid artery was higher in older volunteers. Interestingly, the ratio between carotid and aortic PWV was significantly reduced to 1.0 for older volunteers PWV, representing leveling of wall stiffness. Furthermore, the stroke volume in the internal carotid artery was significantly lower for older volunteers, whereas the stroke volume in the ascending aorta and common carotid artery was not significantly different.

**Table 1:** Properties of pulse wave dynamics of the carotid artery and aortic arch.

	Young	Old	p
Trajectory aortic arch (mm)	99 ± 17	132 ± 17	<0.001
Trajectory left carotid artery (mm)	175 ± 11	173 ± 12	0.65
PWV_aorta_ (m/s)	4.9 ± 0.7	7.4 ± 1.4	<0.001
PWV_carotid artery_ (m/s)	5.7 ± 1.0	6.9 ± 1.5	0.002
Ratio (PWV_carotid artery_/PWV_aorta_)	1.2 ± 0.2	1.0 ± 0.2	0.006
Cardiac output (ml/beat)	94 ± 22	87 ± 12	0.30
Carotid stroke volume			
Common carotid artry (ml/beat)	7.7 ± 1.9	7.7 ± 2.0	0.99
Internal carotid artery (ml/beat)	5.6 ± 1.4	4.7 ± 1.3	0.06
Relative reduction (%)	26 ± 12	39 ± 8	0.002

## Conclusions

PWV leveling between the aortic arch and the left carotid artery at older age represents increased stiffening of the aortic arch and is associated with a reduction in volume flow towards the brain. High-field velocity-encoded MRI is well-suited for PWV evaluation across vascular trajectories.

## Funding

This study is supported by an unconditional grant provided by Dr. Rainer Bluemm (Essen, Germany).

